# Surgical Observations on Tumors, with Cases and Operations

**Published:** 1838-01-01

**Authors:** 


### SURGICAL OBSERVATIONS ON TUMORS WITH CASES AND OPERATIONS. By *John C. Warren*, M.D. &c. &c. &c. Boston, 1837.

[Continued from No. 54. p. 344.]

WE have brought our account of Dr. Warren's work to the termination of the section on Osteo-sarcoma. The seventh section, which succeeds, treats of tumors of the glands, to which we shall proceed without further introduction.

### VII. TUMORS OF THE GLANDS.

Dr. Warren adopts a three-fold arrangement of these tumors, dividing them into, *first*, tumors of lymphatic glands: *secondly*, tumors of secreting glands: *thirdly*, tumors of mucous glands. He is inclined to add, as a *fourth* species, tumors of oleaginous and sebaceous glands.

### 1. Tumors of Lymphatic Glands.

These he considers under the heads of scrofulous, scirrhous, and fungoid.

A. *Scrofulous Glandular Tumors.*—We need say nothing on their characters nor on their history. The only inducement we have for alluding to them, is the opportunity afforded us of noticing the following remarks of Dr. Warren on the use of iodine.

"A medicine," he says, "has been introduced of late years, which has acquired much reputation in this and other forms of scrofulous disease. It is not surprising that physicians should with avidity take up any remedy which may promise to relieve so common and inveterate a disease as scrofula; especially one analogous in its character to those of which experience has most approved. I must say, that after many years trial of the preparations of iodine, in various forms of scrofulous affection, I have rarely seen any very distinct advantages from it. I have employed it in large and small doses, in hospital practice and private, externally and internally. Nor in truth ought we to allow ourselves to expect the results which have been promised. Scrofula is a constitutional, and commonly an hereditary disease. If there be any remedy for such a disease, it must be found in agents which influence the intimate structure of the body more generally and intimately than iodine, or any medicinal substance can do. Such agents are food; a healthy state of the excretory apparatus; a pure atmosphere; and an exercise of the muscular system, suited to the constitution of the patient. I do not wish to dissuade from the use of so convenient a medicine as iodine; but would advise it to be employed in such a way as not to disturb the functions which remain healthy; and that it never should be used to the exclusion of those restorative means, to which reason and experience have given their sanction." 164.

We quite coincide with Dr. Warren in these sentiments. It is unphilosophical and inconsistent with most of what we know in medicine to imagine that any single remedy can possess the powers which have been attributed to iodine, in a constitutional complaint like scrofula. Valuable as many of the new medicines have proved, there have not been wanting hundreds of practitioners who have egregiously over-rated and sadly misapplied them. Because, for example, some forms of scrofula have been benefited by iodine, this has been employed in all cases of scrofula, to the neglect of those old and approved remedial measures, stamped as beneficial by established usage and long-continued general assent.

Some persons would seem to have taken leave of their wits in the treatment of a good many disorders. It is enough for them to get hold of a new remedy, and whether it be iodine or quinine, prussic acid or creosote, it is given in all cases, at all times, and under all circumstances. Such prescribers fancy they possess a special dispensing power in the exercise of reason, judgment, or discrimination. They are not trammelled by any ordinary rules, and their principles, if principles they have, defy vulgar comprehension.

It may be very true, that, because a remedy is new, it is therefore suitable for every body and for every complaint, and it may be true also, that we may prescribe it without troubling ourselves to investigate the disorder, and to judge of the applicability of the drug to the symptoms. We say that this new light may be a good one; but we are so old-fashioned as not yet to read by it. We cannot help thinking, that, after all, a little discrimination is no bad thing, and that our exhibition of new remedies must be guided by principles very similar to those which regulate our administration of old ones. We have not yet found that bark is at once a tonic and a debilitant, a sedative and a stimulant, and it is hardly fair to expect that iodine or creosote should be so. It would be desirable to have an universal medicine, but the belief in one *was* rather a past folly, not a present one. Shall the spirit of the impostors and the dupes of the age of alchemy descend on the doctors of 1837?

The rational mode of determining the properties of any drug consists in first ascertaining what they are in conditions of the body as little removed from health as possible, and then what is their special influence on morbid actions. This being done, we have something like principle to guide us in our further employment of it.

We may ascertain in this manner the positive value of a substance as a remedial agent. But it is necessary to be acquainted with its relative value, that which it possesses in comparison with other remedies. To determine this, an acquaintance with the powers of such other remedies is requisite. Unfortunately many of those, who laud new medicines, are either ignorant of the effects of established ones, or, led away by enthusiasm or by something worse, they most unfairly depreciate them. The consequence is, that new medicines are praised in the most fulsome style for doing that, which medicines in every-day use can do much better. The be-puffed drug has a few months' run, disappoints every body, and is then thrown aside altogether, to make way, possibly for some other drug equally be-puffed, and to be equally forgotten.

It is really lamentable to see the greediness with which the medical public listens to the Katerfeltos, who are daily announcing fresh wonderful nostrums. Disappointment appears to beget, not murder, hope, and the utter failure of one crop of expectations would seem to be the best manure for another. Will wild enthusiasts or crafty rogues *always* take the best guage of the sense of the profession?

We return to Dr. Warren, and to the sober lessons of experience.

B. *Non-malignant Scirrhus of the Lymphatic Glands.*—We have admitted, in the previous article, that the diagnosis of true scirrhus from tumors somewhat removed from scirrhus in malignity, is, in their early stages, and occasionally in all stages, very difficult. We need not rip up the whole question again, but we may observe that, a diagnosis being grounded on a consideration of many circumstances, a wide acquaintance with the laws of malignant and of non-malignant tumors is required.

Scrofulous glandular tumors, for example, usually occur at a certain age, in certain states of body, and run a certain course. A knowledge of these circumstances renders their diagnosis pretty easy. Scirrhous tumors are marked by characters and accompanied with general or local conditions which facilitate, more or less, their discrimination. But other glandular tumors may present themselves, with features far from marked, under conditions not at all distinctive, and to the embarrassment of the inexperienced surgeon. It is probable that such tumors are not of one, but of many kinds, and that their condition is not a fixed but a transition state.

Tumors, for example, form in the neck, resist all treatment for a length of time, and, finally, either subside spontaneously, or under the influence of some medicine. In another case, tumors of apparently a similar description never disappear at all. And, in another, they ultimately exhibit genuine malignant characters. Dr. Warren remarks :—

"The non-malignant scirrhous gland is of a globular form; quite hard; not discoloured nor painful. It is distinguished from the simple scrofulous tumour by its permanent hardness and steady increase in size. Sometimes it is insulated; more frequently double; and not rarely clustered, as in this specimen taken from the axilla, in which you see from fifteen to twenty tumours, suspended from a pedicle of lymphatic vessels. This was dissected from the axilla of a negro boy about eighteen years old. It involved the axillary vein and some of the axillary nerves. The operation was safely terminated, and the patient recovered from it.

When dissected out, this kind of tumour is found to be covered with one or more coats, formed by a condensation of the surrounding cellular texture, and constituting a cyst. On cutting it open, a white substance is seen near its central part, from which issue rays of the same matter to its exterior. Between these rays there is a pale reddish substance, something like the healthy appearance of the lymphatic glands.

The application of the term *scirrhus* to a non-malignant tumour, is objectionable; but there does not appear to be another word to express the hardened lymphatic gland covered by a cyst, and forming a permanent tumour." 165.

What is the surgeon to do with such tumors? The answer appears to be obvious. If the tumour resists all internal remedies and appropriate management, if it increases in size or alters for the worse in character, and if it occasions local or general disturbance, it should, cæteris faventibus, be extirpated by the knife.

c. *Malignant Tumors of the Lymphatic Glands.* Dr. Warren divides these into the scirrhous and the fungoid. He observes, and we concur with him, that "the disposition of lymphatic glands to assume a malignant affection was not so generally admitted some years back, as it is at present. The secreting glands were supposed to be more inclined to come into this state, than the lymphatic. It is however certain that the latter are often the seat of these derangements, without a preceding disorder of the other. The scrofulous habit, which disposes to the simple lymphatic enlargements, is also more frequently the seat of the malignant tumor. The latter, like the other, is found in those parts where the glands are numerous."

If we may trust our own observation, we should say that the scrofulous habit is prone to fungoid but not to scirrhous degenerations. It has long been remarked, that fungoid disease of the eye occurs most frequently in children of a strumous habit. We have noticed, in reference to malignant diseases of the stomach, that if the complexion was fair and the habit strumous, the disease was *generally* fungoid, while scirrhus accompanied a dark complexion and a bilious temperament. Of course these observations must not be taken for more than they are worth; they are rather approximations to the truth than truth itself.

Dr. Warren's description of the characters and march of scirrhus of the lymphatic glands is concise. It begins, he says, with a simple glandular induration, not at first attended with soreness or pain, but exhibiting much hardness in an early stage. After some months, a darting paid is perceived; other glands, in the vicinity, partake of the disease; the patient begins to lose flesh and strength; the glands between the tumor and thoracic duct are enlarged; the pain becomes great, and particularly affects the back, when the tumor is seated in the groin. Watery effusions occur in the thorax, abdomen, and in the extremities; and the patient wastes away under a general disease of the lymphatic apparatus. Not unfrequently the original tumor ulcerates, and produces a disgusting sore attended with great pain. Descriptions of this kind contain no positive diagnostic marks, nor can we wonder at it; there are none.

We pass over some cases, which contain nothing worthy of particular notice, and pause at one distinguished for a remarkable operation. Our readers are aware that, on many occasions, we have earnestly discountenanced severe operations of doubtful utility. We think it is impossible to dwell too often on this subject. Scarce a week elapses without some fresh victim to the Moloch of a morbid thirst for notoriety. This system must be put down. The blood of mutilated and of murdered patients cries loud for retribution.

In submitting the details of the following case to our readers, we must not be understood as reflecting in any degree on Dr. Warren. The case occurred ten years ago, when the nature of the malignant alterations was not so well understood as it is now, and when operations were more freely performed for their removal. It is only within these very few years that great caution has been exercised in resorting to the knife, and that proper principles have regulated its employment. But we will introduce the case.

A farmer, aged 60, of weak constitution, applied to Dr. Warren with a scirrhous glandular tumor in the neck. It had existed for more than a year, and occupied the whole of the left side of the neck, from the ear to the clavicle, and from the trachea to the spine. The mastoid muscle, all the arteries, veins and nerves of the neck, were included in its substance. A process extended under the jaw into the pharynx, and filled the left half of this cavity with a red tumor, and greatly impeded deglutition. The external tumor was very hard, knotted, not discoloured and not tender. The growth had been rapid. The patient's health was failing, and his suffering, from the act of swallowing, quite distressing.

The patient being desirous of an operation, Dr. Warren resolved to be guided by a consultation of the surgeons of the hospital. The result of that consultation was, that the patient should be made acquainted with the danger and uncertainty of a surgical operation, and that, if after a view of these, he desired it to be done, it was right to undertake it. The patient, after a consultation with his friends, determined to go through it, and it was performed at the hospital in the following manner.

"An incision was made from behind the ear to the anterior third of the clavicle; the surface of the tumour uncovered, the mastoid muscle was sought for, but was partly absorbed, and partly buried in the tumour. After clearing the tumour from the ear, the jaw, larynx, and dorsal muscles, an attempt was made to get under the tumour, just above the clavicle; and now the difficulties of the operation appeared. The carotid artery, internal jugular vein, and par vagum nerve, were covered by and connected with processes of the tumour, in such a manner as to render it difficult to distinguish them. This was at last accomplished by breaking down the lower part of the tumour, till these parts were made out. The vein was obliterated, the artery diminished in size, but pervious. A ligature was applied on the latter, and the par-vagum nerve was separated as carefully as possible, though not wholly cleared of the tumour. The separation from the nerves at the upper part of the neck, was next attempted successfully, with the exception of the sublingual, which so barred access to the pharyngeal part of the tumour, that I determined to divide it; and then followed the disease into the pharynx, as far as could be done without cutting open its cavity; the operation was finished by breaking down such parts of the tumour as could not be separated from the nerves. There was but little hæmorrhage, as is common in large, and hard tumours. The parts were brought together, and the patient removed to his bed. He was faint twice, but otherwise supported the operation well." 176.

On the following days, he had great difficulty in swallowing. At the end of a week, this subsided; the wound united by the first intention, excepting the inferior part, which was kept open, and in twenty-two days he left the hospital, and went to his home in the country. A few days before the patient left the hospital, Dr. Warren applied the actual cautery to a portion of the tumor adhering to the parietes of the pharynx. He would have repeated it, but consent was refused. After the lapse of some months, the throat began to ulcerate, and this ulceration gradually extending, produced a difficulty in swallowing, and, wearing him down, proved fatal in about a year from the operation.

It appears to us that this case is a very good example of what, in the present day, we should not do. An aged person has a tumor possessed of true malignant characters, of such a size and in such a situation, that it is unreasonable to anticipate the possibility of removing it completely by an operation. What are we to do? Are we to hold a consultation, represent the case to the patient, and allow him to decide upon the treatment? We say—No. It is very well to say that the case is fairly represented to the patient. The fact is, that it never is nor ever can be represented perfectly fairly. The colouring of the picture is always too light. And, supposing we do represent the case fairly, are we doing justice to the public. It is interested as well as the individual; and, by viewing the case as merely one of personal feeling, we are losing sight of our duty to the community. The proper question to put is this—Is the operation likely to serve the interests of science and of humanity, as well as to flatter the *fallacious* hopes of a timid man? If we feel morally certain that the operation will fail, we should at once discountenance it. The patient is not, cannot be, a proper judge, and the surgeon, who sneaks out of his responsibility under the shadow of a consultation or of a patient's wishes, is not acting the part of a man of science. If the best reasoning in our power tells us that *it is not right* to operate, an operation is unjustifiable.

Dr. Warren proceeds to the examination of fungoid disease of the lymphatic glands. His account of it is not voluminous.

"Fungoides of the lymphatic glands is an insidious, deceitful tumour. When first discovered in the groin, thigh, or neck, it is small; its consistence soft; its connexions confused and not precisely limited. Without colour at first, it sometimes ultimately becomes of a livid red, and when of a considerable size, that for example of a lemon, it has a livid red colour; so that it much resembles an abscess, and, if you had not known the history, you would say it contained a fluid. You puncture it, nothing is discharged but a bloody water or blood. You extirpate it, thoroughly as you think, after some months it appears again in the cicatrix. Do what you will, it proceeds onwards, though with extreme slowness and little pain, and in a course of years gradually affects the lymphatic glands and great organs in the cavities." 187.

The number of lymphatic glands which become disordered, both internal and external, is very great in some cases. They may amount to hundreds. The external glands, which are secondarily affected, are not ordinarily discoloured. It is usually the primary disease, which becomes of a livid red colour. The duration also varies: some of these cases going through their course much more rapidly than others. This is a fact which has struck all observers. It seems, at first sight, strange that a morbid process like the fungoid should now and then remain in abeyance for a lengthened period. But our surprize must lessen, when we reflect that the process itself is probably no more than a modification of healthy action, and that we really know very little about it.

Dr. Warren, if pressed for discriminative characters of the fungoid disease of the lymphatic glands, would say—" the *softness* of the tumor, its tendency to *discolouration*, its *early* influence on the constitution, and the *extent* to which it affects the lymphatic system."

Dr. Warren relates several cases of this disease. They present no very peculiar features, and we pass them by.

### II. Tumors of the Secreting Glands.

The various secreting glands are the seat of the various morbid growths and morbid alterations of structure, in different degrees. Thus, scirrhus, frequent in the mamma, is comparatively rare in the testis: and fungus hæmatodes, common in the latter, is seldom seen in the former. Dr. Warren first treats of:—

### a. *Tumors of the Breast.*

He alludes to the writings of Mr. Travers and of Sir A. Cooper, on this subject, in terms of merited approbation. Sir Astley has described the non-malignant, but reserved to a future opportunity the account of the malignant tumors of the mamma. The non-malignant tumors are enumerated by the worthy Baronet thus—1. The hydatid disease of the breast. 2. The chronic mammary tumor. 3. The cartilaginous and osseous tumor. 4. The adipose tumor. 5. The large pendulous breast. 6. The scrofulous swelling. 7. The irritable tumor. Dr. Warren makes a few remarks on each, and presents some cases. The following are all that deserve extraction.

*Incipient Hydatidal Tumor of the Breast.* This case is worth perusing. Miss ———, in good health, accidentally discovered a small induration in the left breast, in May 1836. It increased considerably from that time to August, when Dr. Warren saw it. The breast was small and flat; and at the first examination scarcely seemed altered from the natural state, but, on comparing it with the other, was found to be decidedly hardened and somewhat enlarged. In a month he saw it again. It had increased considerably at one part, without pain, although she had a slight uneasiness. There was no adhesion to the skin; no discolouration, nor other change in the integuments, excepting that the nipple was somewhat enlarged and the surrounding parts seemed to rise around it, giving the aspect of its being drawn in, although in fact it was of greater dimensions than natural. Not exactly knowing what the alteration was, but fearing that it might, sooner or later, prove carcinomatous, Dr. Warren proposed, and the patient assented to, its removal. The whole of the gland was amputated, leaving a covering of cellular texture over the pectoral muscle. The wound healed by the first intention, and the patient was well in a few days.

"On handling the tumour after its extirpation it was found much firmer than the natural gland: but not scirrhous. The anterior surface presenting nothing remarkable at first view, I turned to the posterior face, and then perceived the soft tumour, whose cavity you here notice, about the size of a nutmeg, and filled with a reddish water. Near to this, were smaller cysts, some of them with opaque, but many with transparent sacs of a yellowish colour, and containing a yellow fluid, which gave them this appearance. Other little eminences were seen covering the whole posterior surface, most of them not larger than the head of a pin and not containing a visible fluid. Again examining the anterior face, I perceived on a slight section that the gland was filled with these little bodies, not larger than a pin's head. The glandular texture appeared to be buried in an adventitious white substance, which obscured its natural aspect and seemed to be a deposit between its lobes, filled with the small bodies abovementioned. The tumour then was constituted by the species of hydatid, called by Sir Astley Cooper, cellulous hydatid, mostly in an incipient state. The sudden increase of swelling was caused by the formation of fluid in the large cyst, which constituted a considerable part of the tumour. Had the disease been left to itself, it would soon have attained a great size." 210.

*Cartilaginous and Osseous Tumor.*—Dr. Warren has not seen this as a distinct disease, but he has witnessed two or three intances of cartilaginous formation, and one of calcareous deposition in the scirrhous breast.

*Scrofulous Breast.*—Dr. Warren details some examples of this affection. But the only case, which we think it necessary to particularize, is not an instance of scrofulous mamma, but of a strumous abscess in the vicinity of the breast, accompanied with a fistulous opening into the lungs, and ultimately cured. The case is related in the words of Dr. Pierson, by whom it was communicated to Dr. Warren.

*Case.*—Miss —— applied to Dr. P., in September, 1831, with a somewhat painful rising on the sternum, just beneath the articulation of the clavicle, which had been several weeks in reaching its size, about two inches in length by one in breadth. The application of leeches, poultices, &c., had no effect to disperse it, and on the 1st of October, the tumor having become softened was opened, and a moderate quantity of thin pus, with some curdy shreds, was discharged. There was a relief of the painful and inflammatory symptoms after the discharge, but the interior of the cavity did not heal, and a fistulous opening was established.

"On the 17th of January, 1832, I dilated the opening in two directions, as the sinus was found to be bifurcated at the top. The lower portion of the sinus healed, while the upper continued to discharge. In this state she visited you on the 7th of February, 1832. It was at this time she commenced using injections of hydriodate of potass, and after they had been thrown into the sinus three or four times, she found that an effort to cough was occasioned, and a portion of the fluid was ejected from the larynx. In order to be perfectly satisfied of this fact, I directed her to use an injection of wormwood, the taste of which she instantly perceived on coughing it up.—There was a gradual diminution of the discharge till the sinus appeared to be very narrow, but still there was constantly a degree of moisture exuding from a minute opening, through which a probe would pass, till it was stopped against the cartilage of the second rib on the right side. This continued to be the condition of it till the month of August, after which I was absent from the country, but the same treament was continued for several months. During a portion of the treatment the external orifice of the sinus was dilated with sponge, by which means a more complete application of the hydriodate of potass was effected. During this period the sinus completely healed. Her general health was at times impaired, and some pulmonary symptoms caused a degree of anxiety, but nothing of a very serious nature occurred, and the only remedies used were a proper attention to diet, exercise, and the functions of the alimentary canal." 224.

Dr. Pierson saw Miss —— on the 2nd October, 1836. The skin was then perfectly healed, and not in the least tender. It adhered in one small point to the sternum, and she occasionally felt some sensations of stricture within the chest.

*Irritable Tumor of the Breast.*—We need scarcely tell our readers that this is a small, hard, sometimes knotted tumor in adult females, married and unmarried, distinguished by its tenderness and disposition to inflame and become painful at intervals, which are sometimes regular. The following case illustrates the disorder very fairly.

*Case.*—A young married lady, with a child seven months old, came to Dr. W. from the country, with a tumor in the right breast, which she had been advised to have removed. She was a delicate person, twenty-two years of age; had formerly been under his care for two or three years, for an affection of the retina, which deprived her of the use of her eyes; but from this she had wholly recovered. At the end of a month after her confinement, she had severe inflammation of the breast, without suppuration, which terminated in leaving a small, hard, tender tumor. At the time Dr. W. saw her, the whole breast was swelled and very painful; with quite a severe constitutional disturbance; quick pulse; great heat; pain in the head; nausea; loss of appetite. Leeches with warm fomentations and purgatives being applied, the swelling of the breast disappeared on the fifth day, and left the small hard tumor as before. She informed Dr. W. that these paroxysms occurred about once a month, though not regularly.—The course advised was to employ leeches and warm fomentations of stramonium in the paroxysms, and in the intervals, to take the sulphate of iron, not exceeding a grain three times a day, and to continue nursing her child. She had only one paroxysm after this, the tumor disappeared, and both the child and herself enjoyed excellent health.

Dr. Warren speaks of a preparation which exhibits the anatomical structure of the tumor. The latter is of an oblong form, about two inches in length, and an inch and a half through. On cutting it open, it displayed a remarkable redness, almost as much as if it had been injected; while the surrounding cellular texture is colourless. The tumor seems to be composed of cellular texture in a condensed state, containing many small bloodvessels. It was taken from a young lady, about twenty years old, who perfectly recovered from the operation, and has remained well since.

We see nothing more in the account of the non-malignant tumors of the breast to occupy us, and we proceed to the malignant. As before, it is only necessary to pick out something here and there. The descriptions are too elementary to permit analysis.

*Fungoid Disease of the Breast* is cursorily noticed.

*Scirrhus.*—The observations on this are more lengthened. Dr. Warren offers an account of the appearances presented by a scirrhous tumor of small size, and at an early stage of its existence, which seems to us to be accurate. Proceeding, he says, from the surface inwards, and scraping away the fat of the breast, a number of white processes are seen radiated from the central nucleus. These at first seem to be mere cords, but on observing further, they proved to be membranous prolongations from the central nucleus to various parts of the breast. Coming to the nucleus itself, this is found to be hard and irregular, and requiring an incision. Cutting through this hard body, we find it grows more firm as we approach its central point, where its hardness is very great. On examining the section thus made, we find the circumference of the nucleus constituted by a membrane or cyst, which has different degrees of distinctness, and disappears at one part where its scirrhous substance is continuous with that of the breast. The substance within the cyst is composed of granulated bodies intermixed with a white mass, of a fibrous aspect and radiated direction. In some instances, the colour of the contents of the cyst is dark. The hard nucleus after a time softens gradually to the consistence of a paste. An absorbtion of a part of it takes place, and in the cavity thus formed there is a fluid of variable colour, either reddish, purple, or black. The nucleus itself he has seen black,—in other words, melanotic.

Mr. Carmichael believes that scirrhus, tubercle, and fungus are all entozoa, or parasitical animals. The scirrhous entozoary formation consists, in his opinion, of three parts:—first, a cyst; second, a softened substance within the cyst—and these two constitute a parasitical animal; third, the scirrhous or cartilaginous formation, which is a barrier thrown up by nature against the further progress of the parasite. It must be owned that the evidence adduced by Mr. Carmichael is far from satisfactory, and that his statements must be deemed, at present, hypothetical.

We agree with Dr. Warren in the general tenor of his observations on the treatment of scirrhus by an operation. If any thing is to be done it must be done early. Though it is too true that there is usually a constitutional vice, and though an operation, under most favourable circumstances, is too often unsuccessful, yet the experience of all surgeons has shewn that occasionally an early removal of the scirrhous tumor is not succeeded by a return of the disease. "Many successful operations," says Dr. Warren, "in cases of undoubted cancer, I have certainly witnessed. Numbers of my patients, operated on ten, fifteen, and twenty years ago, are still living in health, to give their testimony to this statement."

In a previous article, we observed that Dr. Warren appeared to be imperfectly acquainted with the laws and phenomena of those secondary inflammations and collections of matter which have attracted so much attention in England and France. The following observations of our author go to confirm the suspicion that we entertained.

A female was operated on for scirrhus of the mamma. She bore the operation well, and during the following week was in a favourable condition, with the exception of some degree of difficult breathing. In eight or ten days this difficulty increased, the pulse became very quick, the abdomen tumid and painful; and, after three or four days suffering, she died unexpectedly.

On examining the body, the lungs were adherent to the parietes very closely on the right side, and slightly on the left. The non-adherent surfaces were reddened and uniformly covered with a thin fresh layer of lymph. The surface of the pleura exhibited some irregularities, caused by a thickening of the cellular texture under it. The right lung, especially its upper lobe, was in a state of extreme hyperæmia. The left lung was similarly, but more slightly affected. The lower part of the lungs was emphysematous. The mucous membrane of them and of the trachea was inflamed.

The whole surface of the peritoneum was injected and covered with coagulated lymph. The coat of the liver was opaque, its substance crisp. The uterus was enlarged, thickened, very hard, its fundus covered with a hard tumor as large as a walnut. In the substance of the uterus were two or three hard white nodules. These tumors were specimens, no doubt, of "fibrous tumor" of the organ.

This dissection is interesting in this respect. It displayed the condition of the patient at the time of an operation for external malignant disease. Were the irregular thickenings of the cellular texture under the pleura, and the opacity of the coat of the liver preludes to coming malignant alterations of those parts? They wear a suspicious aspect, and shew at all events the dubious state of internal organs in a patient, who, at the time of operation, evinces no symptoms of internal alterations.

But the motive which principally influenced us in noticing this case, was the wish to correct what appears to us a misapprehension on the part of Dr. Warren. The patient died within three weeks after the operation with inflammation of the pleura and the peritoneum, not marked during life by decisive symptoms. These are the genuine characters of the "secondary inflammations." Let us see what Dr. Warren says upon the subject.

"The existence of erythema erysipelatorum in the hospital at the time, gives us reason to believe that this patient was affected and died with an erysipelatous inflammation of the internal organs. No cause we are acquainted with can so well explain this sudden and unexpected termination. Nor is such an occurrence new to me. I have seen numbers of patients perish a few days after operations, at the time that erysipelas prevailed in the hospital, without the slightest external erythema. In such instances, I have been in the habit of stating to you that these patients died of erysipelas, as truly as if they had been covered with an erythematous eruption. The disease is constitutional. It may affect the skin, and generally does so. It may affect the internal organs, without affecting the skin; and in such instances is most dangerous." 256.

It is hardly necessary to comment on these remarks. The idea of internal erysipelas is purely gratuitous and hypothetical. The case corresponds in all respects with that class of cases familiarly known as instances of "secondary inflammations after injuries and operations," and when the phenomena can be readily explained by referring them to that class, it is more philosophical to do so, than to go out of the way for a more dubious causation.

Dr. Warren relates an interesting case of death from admission of air into one of the veins in the axilla, during the removal of diseased axillary glands. However strange the circumstance, it would seem but too certain that it occasionally happens, and that every precaution should be taken by the operator to prevent it. Into those precautions, it would be foreign to our purpose to enter at present.

If we do not misunderstand Dr. Warren, he looks on the fibrous tumor of the uterus as scirrhous. This is very dubious, or, rather, both facts and authority are opposed to such a view. Nothing is more common than to find these tumors in females of advanced years who have presented no symptoms of them during life, and who have died of distinct diseases, without any sign of malignant alterations of other organs. We notice this matter more particularly, because Dr. Warren has built some reasoning upon it.

"Among the inquiries," he observes "in relation to this subject, one would be, whether the uterus is not the organ primarily affected in scirrho-cancer of the breast? It is perhaps more likely to be disturbed by physical and moral causes than the breast; and though disease in the latter organ seems to be excited by local injury in some instances, there are a much greater number where injury of this part does not produce scirrho-cancer. Considering the greater frequency of disease in the uterus than in the breast, is it not probable that where an injury produces disease in this part, the uterus, being previously disordered, may influence the development of scirrho-cancer, by that sympathy which exists between the two organs?" 278.

No doubt the uterus is more frequently disordered, and more prone to disease, than the mamma is, and no doubt the intimate sympathy between the organs must tend to excite morbid actions in the breast. But Dr. Warren's observations tend, if we do not mistake him, to something more positive—tend to giving the uterus the initiative in a large proportion of the scirrhous formations. On this point, if we do not misconceive him, we are compelled to differ from him.

It is a subject of curious, and, could any satisfactory conclusion be arrived at, it would be one of profitable speculation, to examine the causes of constitutional contamination in cases of scirrhus affecting an external organ. Is the scirrhus at first a local and then a constitutional malady?

So far as we can see, the question does not admit of an universal affirmative either way. One patient receives a blow on the breast, a scirrhous tumor is gradually formed, it is removed at an early period by an operation, and no return of malignant disease takes place. Such a case makes for the local origin of scirrhus. But another patient belongs to a family distinguished for a liability to scirrhous growths,* without obvious exciting cause she becomes affected with scirrhus of the breast, she is operated on, and she

* Dr. Warren relates the following example of hereditary tendency to cancer. "The following instances of hereditary cancer have occurred within my knowdies with scirrhus of some other organs. This case makes strongly for the constitutional origin of the malady. An exclusive theory appears inadmissible, and whilst we must confess an extreme degree of ignorance with respect to the mode in which scirrhus is produced, or the tendency to scirrhus formed, we must also confess, that so far as our knowledge goes, it leads to the apparently contradictory conclusion, *that* scirrhus is and is not a true constitutional complaint—is at first merely local in some cases, and from the first constitutional in others. This conclusion reduces us to the necessity of exercising the utmost caution in investigating and in treating each particular case, for on the judgment and discrimination of the surgeon it may depend, whether the patient may not lose a life which an operation would have preserved, or undergo an operation which should never have been performed.

It is consoling to learn, that, according to Dr. Warren, one case in three, within his observation, has been cured by an operation. We fear this is a more flattering estimate of its success than the experience of surgeons in this Country will warrant; but, even with reservation, it is satisfactory.

*Cancer of the Breast in the Male.*—" This disease is rare; I do not recollect more than two cases of it. One occurred twelve years since in a gentleman of this place, about thirty years old. He had not enjoyed good health for three years, being dyspeptic. He had also frequent pains in the chest. These pains at last concentrated in the right side, near the nipple. Passing his hand over this part, he discovered a small tumour. The pains increased and became very severe within six months from the discovery of the tumour. The hardness extended around, involved and drew in the skin, giving it a puckered appearance. The skin ulcerated near the nipple, and discharged a foul matter. I excised the whole scirrhous mass, comprehending the skin with the nipple, and the cellular substance to the pectoral muscle, about four inches in length and two in breadth. The wound of course was long in healing, but has since remained perfectly sound. A few months since I saw him, and ascertained that he was free from pain or trouble at the affected part, and in perfect health." 283.

Tumors about the nipple frequently occur in boys, and are sometimes of long duration. A few leeches are sometimes followed by the disappearance of the tumor. In young infants a tumor also occurs, but subsides spontaneously.

We have seen chronic inflammation of the male breast, in conjunction with venereal tubercle, and yellow ulceration of the throat. It subsided under a mercurial course.

B. *Tumors of the Salivary Glands.*—The sub-maxillary gland, Dr. Warren has found to be frequently enlarged—the parotid rarely—and the sublingual most rarely.

---

ledge, to a family in this vicinity. The grandfather died of a cancer of the lip; whether others of his generation were affected, I know not. The son had a cancer of the breast, and, at the age of sixty, was operated on by my late father, but died of cancer some years after. Two of his sisters had cancer of the breast, were operated on, and ultimately died of the disease. A daughter of one of the ladies had a cancer of the breast, which I removed at an early period; she recovered, but died some years after of disease of the uterus. A daughter of the gentleman has a cancer of the breast, but declines any operation. I have reason to believe that other members of the family are affected, and conceal the existence of the disease."

He mentions a case illustrative of the powers of the imagination, or of—chance. How many a quack and how many a doctor too gets immense credit from the same sort of occurrence.

"Some time since, a female presented herself to me, with a tumour of the sub-maxillary gland, which had become troublesome and alarming to her. It was about the size of an egg, had lasted two years, and was so very hard that I considered any attempt to dissipate it by medicine to be vain, and advised its removal by an operation. To this the patient could not bring her mind; therefore, to satisfy her wish, I directed some applications of considerable activity to be made to the part; and these she pursued a number of weeks, without any change. After this she called on me, and with some hesitation begged to know whether an application recommended to her would in my opinion be safe. This consisted in applying the hand of a dead man three times to the diseased part. One of her neighbours now lay dead, and she had an opportunity of trying the experiment, if thought not dangerous. At first I was disposed to divert her from it; but recollecting the power of the imagination, I gravely assured her she might make the trial without apprehension of serious consequences. A while after, she presented herself once more, and, with a smiling countenance, informed me she had used this remedy and no other, since I saw her; and on examining for the tumour, I found it had disappeared." 295.

Dr. Warren has seen two or three instances of scirrhus of the sublingual gland. He knows of no account, nor do we, of removal of that gland by an operation. In a case in which he removed it, the following steps were adopted. The patient being seated in a chair, the mouth kept open by a wedge, the gland was griped by a double-pointed forceps, and an incision the length of the gland made from behind forwards on each side. The knife then passed behind the posterior extremity of the gland would have brought it out at the next incision, but the patient made a violent effort to throw out the blood, which compelled the operator to stop. Then, laying down the knife, the excision was completed by a stroke of the scissors. The bleeding was less than was expected. No arteries were tied. The wound healed in about two weeks.

There is nothing on the *Tumors of the Thyroid Gland* to detain us. The same remark applies to the chapter on *Tumors of the Testis.*

c. *Tumors of the Mucous Glands.*—Under this general head, Dr. Warren considers the tumors of the lip, tongue, and intestinal canal, of the bladder, vagina, uterus, and penis—that is, of the mucous membrane of these parts. His observations and conjectures on the subject are summarily contained in the following brief passage.

"Many of the tumours situated on the mucous membrane, originate in the glands scattered through their course. Perhaps all the cancerous diseases of this texture take their origin in these parts. The early stage of such affections exhibits a white fibrinous* substance, either intermixed with muciparous glands, or lying on their surface; and the glands themselves are much enlarged in the vicinity of the tumour. Even we can discern, by the microscope, spiculi, of whitish substance, extending from them, and running into the fibrinous induration, or from the indurated texture to the glands. There is therefore, it seems,

---

* "By fibrinous substance, I mean a texture formed of, and resembling the fibrin of the blood. Fibrous substance I understand to mean the white, insensible, inelastic texture of tendons and aponeuroses."

a deposite of new substance from the disordered glands, which forms the beginning of these tumours. This deposit goes on to a limited extent; and in a longer or shorter time, ulceration begins, commonly at the most superficial part. Then an ill-looking excrescence is protruded; and thus ulceration and deposition go on together, in a slow manner. We can easily conceive of the progress of increased deposition from some local irritation, as tobacco, applied to a part covered by a thin cuticle. But what brings on this process of ulceration, is not so easy to explain.

The early stage of this disease is not accompanied with pain. When ulceration begins, there is a burning and shooting sensation. After the disease has continued for some time, the lymphatic glands, in the course of the absorbent vessels, become swollen, and the disease may then be considered as incurable." 340.

There can be little doubt, that the mucous glands are the most frequent seat of the tumors which form in or spring from mucous membranes. But such tumors are certainly developed at times in other portions of the tissue.

Dr. Warren says nothing of consequence on *Cancer of the Lip*.

*Tumors of the Tongue.* Dr. Warren observes that there is an enlargement of the mucous glands of the tongue, formidable in appearance, but not malignant. These glands become quite prominent; of a strong red colour; are seen most commonly in the lateral parts of the organ; and present an appearance which might be mistaken for that of a fungous tumor. On examination, it will be found, in these cases, that, the patient having been out of health, some local cause, as a broken tooth, has irritated the tongue, and produced the appearance mentioned. The part is sensitive; not painful; there is no real ulceration nor thickening of the tongue: by which circumstances the disease is distinguished from cancer.

After giving a case which is incomplete, he goes on to remark, that, the large mucous glands on the surface of the tongue, near its root, are sometimes swollen from a derangement of the stomach. This condition may continue for months, but at length gives way to a proper regulation of the stomach and bowels. It is sometimes useful to make a local application of the solution of nitrate of silver.

The glands at the back part of the pharynx, behind the fauces, and also those lower down behind the larynx, are subject to a similar affection. Those in the last situation, being seated in the contracted part of the pharynx, cause much inconvenience, and are least tractable. Neither of these affections are of a scirrhous nature.—The same course of treatment is proper in both. When the nitrate of silver is applied in the last situation, it may be done by the sponge of a probang, dipt in the solution.

The tongue, and the lips, and the mucous membrane of the mouth, are subject to a small blue-coloured rising, which is constituted by a cyst, containing a transparent, glairy fluid. This often subsides of itself; sometimes is cured by a puncture, to discharge its contents; and sometimes requires excision.

These statements coincide perfectly with our own experience. We would add that a kind of chronic inflammation of the mucous glands at the root of the tongue and of those on the posterior wall of the pharynx is not uncommon in persons who have had secondary syphilitic ulceration of the throat, or who have undergone a protracted course of mercury. On looking into the fauces the mucous glands are perceived to be enlarged and red, and usually a certain quantity of muco-purulent secretion is seen adhering to the back of the pharynx. In conjunction with these appearances, the mucous membrane both of the tongue and of the pharynx is not uncommonly denuded more or less of its epithelium, and an incautious or ill-informed person may and often does mistake the affection for secondary ulceration of the parts.

The treatment of these cases consists in regulating the secretions—in improving the general health by mild tonics, proper diet, change of air—and in ringing the changes on astringent local applications, including the use of the nitrate of silver. The complaint is troublesome and tedious, but it has always, in our experience, been ultimately cured.

Passing over *Cancer of the Tongue*, to our knowledge of which Dr. W. does not sensibly contribute, and *Scirrhus of the Soft Palate*, we may introduce a case of tumor of the pharynx, remarkable for the hazardous but successful operation performed for it.

*Case.* "In the month of June, 1835, my attention was called to a disease of this kind by Dr. Shattuck. A patient of his, Mrs. Aves, sixty years old, had been labouring under a scirrhous tumour, on the left side of the pharynx, for some years. When I saw it, the fauces were nearly filled, respiration was impeded, and deglutition difficult. Her pulse was quick, tongue furred, appetite poor, and she had the aspect of a person sinking under the pressure of disease. I was disposed to decline any operation, from the little hope of a radical cure, and the great danger of immediate death. Dr. Shattuck urged that she must die with the disease, and that, if the removal of the tumour did not effect eradication, it yet might give some relief from the distressing state in which she was. These reasons influenced me to perform the operation immediately. The jaws being fixed, the hard tumour was seized with a pointed forceps, and, with a round-edged knife, I removed the mass by two strokes of the instrument. An enormous gush of blood followed. Passing the finger in, I felt another mass in the pharynx, below the first, which was seized, and cut out by a curved probe-pointed bistery. When the hæmorrhage had a little subsided, a red-hot iron was passed, on a conductor, to the diseased spot twice. No further hæmorrhage occurred." 357.

Some applications of caustic potass were subsequently necessary, and an exfoliation of a small piece of the superior maxillary bone followed. The patient slowly recovered, but died in the Winter of 1836 of an accidental peritonitis. The tumor was partly of a cartilaginous consistence, and contained calcareous matter.

We cannot say that we approve of operations for malignant disease on tumors in such situations. When we put together the immediate risk, the uncertainty with respect to the complete extirpation of the morbid mass, and the probabilities of a future return of the complaint, we offer, we think, sufficient reasons for prudent surgeons to hold their hands.

In speaking of *Enlarged Tonsils*, Dr. Warren recommends for their excision an instrument resembling that of Dr. Physick. It is something like a guillotine in miniature,—a convex knife, sliding in a grooved ring of a diameter proportioned to the size of the tumor, from an inch downwards. Being passed into the mouth, it compresses the tongue; the ring is slipped over the tonsil; the knife is pushed down, and cuts it off. The whole tonsil is not removed in this operation. This is impracticable and unnecessary.—It is proper to have at hand the means of applying the actual cautery, should the severity of hæmorrhage require it. Dr. Warren recommends the operation in cases of high inflammation of the tonsil.

Some cursory observations are made on tumors of the mucous glands of the stomach and intestines. The only passage which requires notice is Dr. Warren's account of "scirrhous cancer" of the rectum, a disease rare in comparison with the stricture produced by scirrhous parietes of the canal. It is seated most frequently, according to our author, at the back part of the cavity, towards the os sacrum. It begins with a roughness of the intestine, like a cluster of enlarged mucous glands. It soon makes itself known by a pain in evacuation, which increases with the growth of the tumor, and at last becomes excessive. In the early stage there is no discharge but of blood. After it has attained a considerable size, a discharge of mucus and occasionally of a little pus appears. The pain becoming constant, and very distressing on evacuations, the patient is obliged to resort to full doses of opium. The disease is always fatal.

*Tumors of the Mucous Glands of the Vagina.*—Enlargements, says our author, of the mucous glands occur most frequently in females of delicate and scrofulous habit. They appear scattered through the parietes of the canal, in the form of papillary eminences, of various size, from that of a millet seed to that of a pea. When very small, no distinct symptoms indicate their existence, excepting there is an actual examination by touch. When of large size, an uneasiness exists in the passage; a slight discharge; paroxysms of strangury; and a distinct tenderness on touching them.

In illustration of the preceding description, he relates a case.

A married lady, aged 22, who had had no children, complained of an uneasiness in the urethra, with a bearing down, frequent attacks of strangury, a constant sense of heat in the vagina, and a mucous discharge, sometimes tinged with blood. On making an examination with the finger, the parietes of the vagina were found studded with enlarged glands, of various sizes. When pressed, these were painfully affected, the larger more than the small. Her health was tolerably good otherwise. The menstrual evacuations were regular.—She was advised to assume the horizontal posture, use a warm injection of soap and water twice a day, apply six leeches to the perineum every five days, and avoid the use of animal food. This treatment was continued many weeks, without change in the symptoms. At the end of four months, the burning diminished. It was not till after seven months' steady pursuit of the course, that the strangury and heat in the affected part ceased. A discharge continued two or three weeks longer. The examination at the end of this time was without any uneasy sensations; the tumors had disappeared; and she has recovered perfect health, and retained it for some years.

Some brief descriptions of *Tumors of the Uterus*, and of *Cancer of the Penis* complete the Section devoted to tumors of the glands. With the termination of that Section we also terminate this article. As we observed, it could not be analytical, because in a work professing to record experience, much must be familiar, and what is new or striking is necessarily unconnected in detail. Dr. Warren enables us to bring before the English public a not-unfavourable view of American surgery, and in that point of view, if in no other, he would lay us under obligations to him. In our next Number we shall conclude our account of the work, and we hope we shall have conveyed to our readers some valuable remarks of the author, some important facts, and some useful reflections on a subject which is attracting and must ever attract the attention of the profession—the morbid growths to which the body is liable.

